# Fertility Preservation in Benign Gynecological Diseases: Current Approaches and Future Perspectives

**Published:** 2019

**Authors:** Zaki Sleiman, Erbil Karaman, Milan Terzic, Sanja Terzic, Giovanni Falzone, Simone Garzon

**Affiliations:** 1- Lebanese American University, Department of Obstetrics and Gynecology, Beirut, Lebanon; 2- Department of Obstetrics and Gynecology, Medical Faculty, Yuzuncu Yil University, Van, Turkey; 3- Department of Obstetrics and Gynecology, National Research Center of Mother and Child Health, University Medical Center, Astana, Kazakhstan; 4- Department of Medicine, Nazarbayev University, School of Medicine, Astana, Kazakhstan; 5- Department of Obstetrics, Gynecology and Reproductive Sciences, University of Pittsburgh, School of Medicine, Pittsburgh, Pennsylvania, USA; 6- Obstetrics and Gynaecology Unit, Umberto I Hospital, Enna, Italy; 7- Department of Obstetrics and Gynecology, Filippo Del Ponte Hospital, University of Insubria, Varese, Italy

**Keywords:** Benign ovarian tumors, Counseling, Endometriosis, Fertility preservation

## Abstract

Although fertility preservation is a growing topic in the management of oncological diseases, different benign gynecological pathologies are able to compromise the ovarian reserve due to mechanisms related to the pathology itself or secondary to the performed treatments. Endometriosis, benign ovarian tumors, adnexal torsion, familiarity and genetic syndromes are all benign conditions that can compromise the ovarian reserve. Endometriosis and particularly endometriomas provide a direct damage to ovarian reserve, with different mechanisms, and an indirect damage related to surgery. Similarly, benign ovarian tumors can provide a detrimental effect on ovarian reserve for the surgical treatment, especially for bilateral or recurrent tumors, and in case of secondary adnexal torsion with late diagnosis. Different fertility preservation options are available and should be considered particularly in cases with bilateral or recurrent pathology and/or surgery. In general, the identification of patients at risk of early ovarian failure, for benign gynecological disease or based on known genetic causes or familiarity, is of paramount importance in order to apply fertility preservation techniques before the complete depletion of ovarian reserve.

## Introduction

Fertility preservation should be taken into account and proposed also to patients suffering from benign diseases which can lead to an early reduction of the ovarian reserve and consequent premature ovarian insufficiency (1, 2). The ovarian reserve is defined as the quantity and quality of the primordial follicle population that develops from 100 to 200 *germ* cells (3). These cells, starting from the first weeks of embryonic development, undergo a rapid proliferation reaching a peak of a few millions of primordial follicles around 18–22 weeks. 85% of this population is lost before birth with a decline that continues, with variable grade, throughout the reproductive life. From puberty to menopause, only about 450 follicles develop until ovulation. Therefore, the main mechanism that determines the physiological age-related decline of ovarian reserve is follicular atresia, with about 1500 primordial follicles available at menopause (4). Although fertility preservation is a growing topic in the management of oncological diseases (5–6), different benign gynecological pathologies are able to compromise the ovarian reserve due to mechanisms related to the pathology itself or secondary to the performed treatments. Endometriosis, benign ovarian tumors, adnexal torsion, familiarity and genetic syndromes are all benign conditions that can compromise the ovarian reserve. All of them require an estimation of the impact that they and any possible treatment have on the woman’s reproductive window (1, 2).

### The impact of endometriosis on the ovarian reserve and strategies to preserve it:

Accumulating evidence suggests that infertility is often associated to endometriosis, and different possible mechanisms were proposed based on the location and extension of the disease (7, 8). Endometriosis consists in the presence of functional endometrial-like tissues (glands and stroma) outside the uterus. It is related to pelvic pain and subfertility in reproductive age, can severely compromise the quality of life of affected women (9–14) and may require extensive surgery (15–17). It is a chronic inflammatory estrogen-dependent pathology inducing chronic inflammatory response and damage-repair mechanisms with subsequent scar tissue and adhesions that are able to distort women’s pelvic anatomy (9, 10, 18, 19). Ovarian localization of endometriosis is the main factor influencing the ovarian reserve. From the earliest stages of endometrioma development, a reduction with focal loss of primordial follicles concomitant with a loss of the cortical stroma has been demonstrated (20). The ovarian cortex plays a fundamental role in the sustainment of ovarian reserve supplying the follicles with nourishment, mediators and somatic cells for follicular growth (21). The presence of endometrioma determines the establishment of a local inflammatory process that is involved in the destruction of the ovarian cortex with subsequent loss of cortical stroma due to fibrosis and neovascularization with, finally, a depletion of primordial follicles. Additionally, follicular depletion is related to a direct damage by inflammatory mediators and oxidative stress that causes apoptosis and necrosis of the follicles. This depletion determines a local reduction of AMH levels with subsequent increase of follicle recruitment and secondary local atresia. Moreover, the protracted distension of ovarian cortex due to the presence of the cyst seems to play a further detrimental role (21–23). Endometriosis is a complex pathology with unclear etiopathogenesis and different involved mechanisms such as apoptosis, angiogenesis, inflammatory microenvironment, and oxidative stress that are not only a possible cause of ovarian reserve reduction but even a cause of reduced oocyte quality (24–31). Nevertheless, the potential impact of endometriosis on oocyte quality is debated in relation to the conflicting results between the reduction of fertilization, implantation and clinical pregnancy rate, compared to a lack of evidence on reduction of live birth rate (32). Although these clinical data are not completely consistent with laboratory data reporting a quantitative damage to ovarian reserve, the impact of endometriosis is in general considered deleterious (33).

The endometrioma does not respond to medical therapy. Therefore, laparoscopic surgical cystectomy is currently the treatment of choice that should be considered for endometriomas with a minimum diameter over 4 *cm* (34). Nevertheless, surgical treatment for endometriosis, and particularly for endometriomas, has been related to the damage of ovarian parenchyma and related ovarian reserve. Postoperative AMH levels were significantly reduced (35), ovarian function tests severely compromised 5–10% of patients who had underwent surgery (36), ovarian response to hyperstimulation for IVF appears to be halved (37), with a low but consistent risk of post-operative ovarian failure (38). Although a recent meta-analysis reported no difference in the antral follicle counts, the surgical procedure for ovarian endometrioma should be considered harmful for the ovarian reserve, because it alters other variables reflecting the ovarian function such as a lower level of AMH and a higher dose of gonadotropins needed for a subsequent ovarian stimulation (33, 39). Different surgical approaches have been proposed comparing laparoscopic and laparotomic approach, electro-surgical haemostasis and suture, stripping and drain-age with laser vaporization or a combination of them. Although some of them seem to be less harmful to the ovarian reserve, ovarian damage cannot be excluded (33, 40). In preservation of fertility in a patient affected by endometriosis, many factors should be considered; the potential damaging surgical treatment, the reduction of ovarian reserve secondary to the endometriosis itself, the chronicity, the tendency to relapse after surgery, and the incidence of pathology are the typical ones (33, 41, 42). Different techniques are available for the preservation of fertility (1, 33, 43). The cryopreservation of oocytes and embryos is the consolidated technique with the benefit of oocytes cryopreservation that provides autonomy to women in relation to the long-term preservation (44–45). With regard to these techniques, greater efficacy is demonstrated if preservation is performed before the age of 35 years. These data should be carefully generalized and are directly related to the experience of different centers (46). Cryopreservation of the ovarian tissue is the only available technique for prepubertal girls and women who cannot delay therapy. It should be considered, although currently experimental, for a possible future clinical implementation even for benign diseases (1, 47, 48). There is evidence that cryopreservation of ovarian tissue can also be indicated in patients with endometriosis because healthy tissue removed with the endometrioma can be isolated and cryopreserved (33, 43, 49). Based on currently available knowledge, the preservation of fertility should be considered in patients with endometriosis at high risk of bilateral ovarian damage, bilateral endometriomas, recurrence of mono or bilateral endometriomas with previous surgery for bilateral or contralateral endometriomas. In these conditions, although the quality and quantity of recoverable oocytes vary, the probability of using them is very high. In the case of unilateral endometriomas or severe endometriosis not involving the ovaries, the role of preservation of fertility is more limited and debated (33). Moreover, the age of patients should also be considered.

When the first surgery is performed at a younger age, the recurrence rate has higher odds. In such cases, fertility preservation techniques are of utmost importance especially because another surgery may be eventually needed (33, 50, 51). In addition, other factors such as familiarity with early ovarian failure, BMI, smoking, alcohol, and ovarian reserve markers should be considered (52). Overall, although endometriosis could be considered an indication for fertility preservation, primarily by oocytes cryopreservation, additional clinical and cost-benefit data are needed before a routine application is implemented. Moreover, both assisted reproduction techniques and endometriosis are risk factors for ectopic pregnancies (53–54). Efficacy data related to fertility preservation techniques in endometriosis patients are limited, and a cost-benefit analysis is required in relation also to the high incidence of the disease. It is possible that the search for early offspring in patients with partner has a better cost-benefit ratio as compared with the postponement allowed by the preservation of fertility that requires the access to the techniques of assisted reproduction. Finally, the possible use of estrogen-progestin therapy after the first surgery reduces the risk of recurrence without reduction of pregnancy rate (33, 43, 55).

### Benign non-endometriotic ovarian cysts and adnexal torsion:

Benign ovarian tumors represent a frequent and heterogeneous gynecological pathology. The correlation between infertility and benign non-endometriotic ovarian cyst is debated. A possible effect is related to the mechanical distension of ovarian cortex for the endometrioma (23). Nevertheless, the ovarian damage is mainly due to surgery especially for bilateral or recurrent tumors and in case of secondary adnexal torsion with late diagnosis (56–57). Adnexal torsion is often associated with adnexal tumors but it may also occur in a normal ovary, probably due to excessive ligamentous laxity, tubal spasm or more frequent intra-abdominal pressure changes in prepubescent and neonatal age (58).

Despite the fact that there is no uniform standard to assess the viability of the ovary and choose conservative surgery or radical surgery for patients with adnexal torsion, patients with ovary/ovarian cyst torsion can attempt to preserve the ovaries without serious clinical complications such as abdominal infection or thrombotic diseases (59). In this regard, adnexal detorsion is safe and essential for the preservation of fertility due to the high recovery rate of ovarian function, especially in pediatric population (60). The detorsion must be associated with the immediate or subsequent removal of the tumor/cyst or with the ovaropexy in order to prevent recurrence (1, 58). Laparoscopic conservative surgery should be considered even in rare conditions, such as ovarian ectopic pregnancy (61, 62), which can occur in adolescence (63) and more often after IVF (62, 64).

Fertility preservation techniques for benign adnexal tumors and adnexal torsion should be considered in the case of bilateral ovarian surgery or adnexal torsion, bilateral or contralateral repeated torsion or ovarian surgery, unilateral adnexectomy with single residual ovary for suspected malignant tumor or necrotic adnexa secondary to protracted torsion (1, 2). Fertility preservation techniques available for the patient in fertile age are the cryopreservation of oocytes or embryos with the same consideration for endometriosis (44–45).

Of note, ovarian stimulation for fertility preservation in these patients, and in patients affected by endometriosis, requires attention, and is difficult to standardize.

It is important to evaluate whether these patients should be considered as poor responders due to the reduced ovarian reserve secondary to the pathology or the subsequent surgery. Therefore, a personalized approach is of paramount importance (65–67). Moreover, strategies aimed to improve the outcomes of ovarian stimulation could be suggested, particularly if concomitant pathologies are present, such as polycystic ovarian syndrome (68–75). Although cryopreservation of ovarian tissue is the only technique for the prepubertal age, it can also be applied in the case of healthy ovarian tissue removed concomitantly with the removal of benign tumors in the woman of childbearing age (76).

### Premature ovarian insufficiency and Turner syndrome:

Premature ovarian insufficiency has a prevalence of about 1% in the female population and is defined as the loss of ovarian function and contextual ovarian reserve before age 40 (77). There are numerous possible non-ovarian causes including autoimmune diseases, surgical therapy, chemotherapy and radiotherapy. Conversely, an intrinsic defect of ovarian and follicular function is found in genetic conditions including Turner syndrome, fragile X syndrome, other chromosome X depletion or mutations, different autosomal genes, and metabolic diseases such as galactosemia. Although the genetic causes are increasingly evident, the idiopathic cause is the most frequent (77, 78). Turner syndrome, characterized by the X chromosome monosomy, is one of the main causes of early ovarian failure. This syndrome with incidence of 1: 2500 female newborns is characterized by accelerated apoptosis of germ cells before puberty with depletion of the ovarian reserve before 10 years (79). A variable phenotype is often present also in relation to the ovarian function due to the possible mosaicism linked to the coexistence of a cellular population 45,X and 46,XX or other condition with variable X chromosome impairment (79). Of note, in Turner syndrome, it is of paramount importance to evaluate the general and cardiological condition of the patient to exclude any contraindication to pregnancy.

In general, in patients with established ovarian failure, regardless of the etiology, fertility preservation techniques cannot be applied with the exception of egg donation (3, 7). Therefore, it is necessary to identify patients at risk of early ovarian failure, based on known genetic causes or familiarity, in order to apply fertility preservation techniques before the complete depletion of ovarian reserve (2, 77). The available techniques to preserve fertility have variable success and are closely linked to the pubertal state, to the residual ovarian reserve and to the psychological development. In women in fertile age, oocyte cryopreservation should be considered the first choice. Conversely, cryopreservation of ovarian tissue, which remains an experimental technique, is the only available method for the prepubertal patients, and it should be applied even at a very young age (1–2). In early ovarian insufficiencies in which there is a need to preserve pre-puberty, particularly Turner syndrome, it is important to emphasize that the preservation of ovarian tissue associated with reimplantation or *in vitro* oocytes maturation represents an experimental technique. Moreover, the risk of transmission of the genetic syndromes, the available preimplantation genetic testing, and the ethical implications remain to be discussed (79).

## Conclusion

Even patients suffering from benign gynecological diseases can experience a failure of the ovarian reserve, that can be related to both the specific pathology or the available treatments. An adequate estimation of potential ovarian impairment is fundamental in order to consider and plan a strategy for fertility preservation (1, 2). Fertility preservation techniques available do not differ from the techniques used for cancer disease (1, 2). However, the evidence is still limited, and further investigations are required in order to clearly define the indications to implement fertility preservation techniques in the benign gynecological pathologies.
